# Amylose primitive médiastinale d'aspect pseudotumoral

**DOI:** 10.11604/pamj.2015.20.449.6248

**Published:** 2015-04-30

**Authors:** Madiha Mahfoudhi, Habiba Mamlouk, Sami Turki, Adel Kheder

**Affiliations:** 1 Service de Médecine Interne A, Hôpital Charles Nicolle, Tunis, Tunisie

**Keywords:** Amylose AL, adénopathies, médiastin, AL amyloidosis, lymphadenopathy, mediastinum

## Abstract

L'amylose primitive médiastinale isolée est rare et de diagnostic difficile. Nous rapportons l'observation d'un patient âgé de 41 ans ayant présenté une dyspnée et des crachats hémoptoïques. A l'examen physique il n'avait pas d'hypotension orthostatique. Les aires ganglionnaires périphériques étaient libres. La tomodensitométrie thoracique a objectivé un magma d'adénopathies médiastinales réalisant une masse de 45 mm x 60 mm. L'examen anatomopathologique d'une biopsie ganglionnaire guidée par médiastinoscopie a conclut a une amylose médiastinale de type AL. Il n'avait pas d'autres localisations amyloïdes. Un myélome multiple a été éliminé. Le diagnostic d'amylose primitive médiastinale de type AL a été retenu. Le traitement s'est basé sur des cures de Melphalan-prednisone. La chirurgie était évitée vu le risque hémorragique élevé. L’évolution était marquée par l'amélioration de la dyspnée, la disparition de l'hémoptysie et la diminution de la taille de la masse ganglionnaire devenant 25 mm x 20 mm.

## Introduction

L'Amylose correspond à un dépôt extracellulaire d'une substance amyloïde ayant des particularités tinctoriales et structurales spécifiques. Une forme ganglionnaire pseudotumorale connu sous le nom d'amyloidome, quelque soit sa localisation, est rare et peut être compressive [1]. L'amylose médiastinale est extrêmement rare. Elle n'est pas souvent prise en considération dans le cadre de diagnostics différentiels des patients présentant une symptomatologie respiratoire. Aucun signe clinique, biologique et radiologique n'est spécifique du diagnostic [1–3]. L'examen histologique d′un échantillon obtenu par biopsie au cours d'une thoracotomie ou guidée par thoracoscopie avec réalisation des colorations spécifiques confirme le diagnostic d'amylose. L'analyse immuno-histochimique permet de réaliser le typage d'amylose (AA, AL,…) [3, 4]. Le but de ce travail est de montrer à travers cette observation et les rares cas publiés dans la littérature la difficulté diagnostique en cas d'un amyloïdome médiastinal isolé responsable d'un retard du diagnostic positif et de la prise en charge thérapeutique.

## Patient et observation

**Figure 1:**
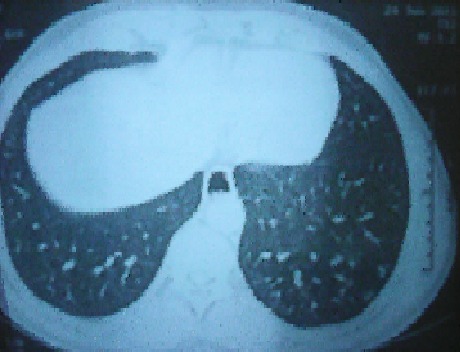
TDM thoracique montrant un magma ganglionnaire formant une masse médiastinale de 45 x 60 mm

**Figure 2:**
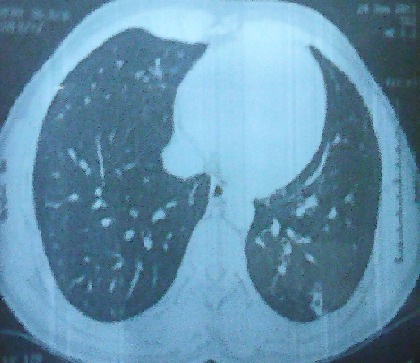
TDM thoracique montrant une diminution de la taille de la masse médiastinale (25 x 20 mm)

L'histoire remonte au mois de février 2010 marquée par l'apparition, chez un homme âgé de 41 ans sans antécédents particuliers, d'une dyspnée d'aggravation progressive et des crachats hémoptoïques, indiquant la réalisation d'une radiographie de thorax qui a objectivé une opacité médiastinale supérieure. Il a été hospitalisé pour exploration de cette opacité. Il n'avait pas de fièvre, ni d'altération de l’état général. A l'examen physique il n'avait pas d'hypotension orthostatique, ni macroglossie, ni gros nerf cubital. Les aires ganglionnaires étaient libres. A l'examen biologique, les fonctions rénales et hépatiques étaient normales. Il n'avait pas d'anomalies sur la numération formule sanguine, ni de syndrome inflammatoire biologique. La tomodensitométrie thoracique a objectivé un magma d'adénopathies confluentes pré et latéro-trachéales droites réalisant une masse de 45 mm de grand axe transversal étendue sur 60 mm de hauteur (Figure 1). Cette masse refoule et déforme en arrière la paroi trachéale antérieure, la veine cave supérieure qui reste perméable en dehors. Elle arrive au contact de la paroi interne de l'aorte horizontale avec disparition du liseré graisseux de séparation. Les échographies cardiaque et abdominale étaient normales, ainsi que le scanner abdomino-pelvien. Par ailleurs, la fibroscopie bronchique avec biopsies étagées et le lavage broncho-alvéolaire réalisés à la recherche d'une origine néoplasique étaient sans anomalies. La recherche de *Mycobactérium tuberculosis*, l'intradermo-réaction à la tuberculine et le Quantiféron test étaient aussi négatifs. L'examen anatomopathologique d'une biopsie ganglionnaire, guidée par médiastinoscopie, était déterminant concluant à une amylose médiastinale de type AL (kappa) avec absence de signes de malignité. Par ailleurs, l’étude de la biopsie labiale ainsi que rectale n'a pas retrouvé de dépôts amyloïdes. Un myélome multiple a été recherché. L'immunoélectrophorèse des protides dans le sang n'a pas détecté d'immunoglobuline monoclonale. La protéinurie de Bence et Jones était négative. Le dosage de béta 2 microglobulinémie n’était pas élevé. Le bilan radiologique osseux standard ainsi que l'IRM rachidienne n'ont pas révélé d'image lytique ou de tassement. La ponction sternale et la biopsie ostéomédullaire ont montré une moelle de richesse normale. Le diagnostic d'amylose primitive médiastinale de type AL a été retenu. Le traitement s'est basé sur des cures de Melphalan-prednisone. La chirurgie était évitée vu le risque hémorragique élevé. L’évolution était marquée par l'amélioration de la dyspnée, la disparition de l'hémoptysie et la diminution de la taille de la masse ganglionnaire devenant 25 mm x 20 mm (Figure 2). Le recul évolutif est de 4 ans.

## Discussion

L′amylose est un groupe hétérogène de maladies caractérisé par le dépôt de diverses protéines ayant un aspect fibrillaire avec une conformation ‘ plissée. Elle est classée comme primitive ou secondaire, et systémique ou localisée. L'amylose systémique correspond principalement à trois types, l′amylose en rapport avec la présence de chaînes légères d′immunoglobuline, l′amylose à transthyrétine et l′amylose AA. Le diagnostic différentiel est fait par l′examen immuno-histochimique. Les manifestations cliniques dépendent des sites du dépôt amyloïde. Au cours d'une amylose systémique, l'atteinte thoracique est principalement cardiaque avec un risque élevé de mortalité [1, 2]. Une atteinte trachéo-bronchique est rarement isolée ou survient surtout le cadre d'une atteinte systémique. Par contre, une amylose localisée à un organe peut avoir un aspect pseudotumoral correspodant à un amyloïdome. Ce dernier est le plus souvent de type AL. Une amylose ganglionnaire peut se manifester sous la forme d'un magma donnant un aspect d'amyloïdome mimant une métastase ganglionnaire ou un lymphome. Un amyloïdome médiastinal est une maladie extrêmement rare qui peut être latente pendant des mois. Fiorelli A et al ont présenté le cas d'un amyloïdome médaistinal antérieur avec un patient complètement asymptomatique [3]. Le tableau clinique est le plus souvent symptomatique. Il peut se révéler par une dyspnée d'aggravation progressive, une toux réfractaire, une gêne ou une douleur thoracique et une hémoptysie d'abondance variable. La radiographie standard du thorax montre un aspect non spécifique correspondant à une opacité homogène ou hétérogène, de limites et de taille variables. La tomodensitométrie thoracique est un examen capital puisqu'elle montre la présence d'un magma ganglionnaire médiastinal. Elle détermine la taille et les rapports de cette masse pseudo-tumorale avec les vaisseaux et les organes avoisinants. Ces détails sont essentiels pour la décision thérapeutique. Dans une série de trois cas d'amylose ganglionnaire du médiastin, la tomodensitométrie a montré un processus tumoral médiastino-pulmonaire dans deux cas et un manchon tissulaire péricarinaire dans le troisième cas [5]. Matsuguma H et al ont publié un cas d’élargissement médiastinal associé à des calcifications dont le diagnostic étiologique a conclut à une amylose [6].

Une fibroscopie bronchique retrouve parfois un épaississement bronchique évoquant une amylose trachéo-bronchique associée. L’étude des biopsies bronchiques étagées avec coloration au Rouge Congo permet de confirmer le diagnostic d'amylose, de typer l'amylose et d’éliminer une néoplasie. Notre patient n'avait pas de localisation trachéo-bronchique associée. Dans la publication de Ridène et al concernant 3 patients ayant présenté un amyloïdome médiastinal, la fibroscopie bronchique a montré une muqueuse infiltrée simulant une lymphangite carcinomateuse dans un cas, elle était normale dans les deux autres cas [5]. Le diagnostic est aisé en présence d'une ou de plusieurs atteintes amyloïdes extra-médiastinales. Les biopsies labiales, rectales ou de la graisse sous cutanée abdominale sont les plus rentables pour le diagnostic d'une amylose systémique. Ainsi, Moles MP et al ont présenté le cas d'un amyloïdome médiastinal associé à un amyloïdome abdominal, une atteinte cardiaque, rénale et médullaire, il s'agissait d'une amylose AL primitive [7]. Une amylose médiastinale solitaire sans autre localisation amyloïde constitue une difficulté et un retard diagnostique conditionnant le pronostic. Plusieurs diagnostics différentiels doivent être évoqués devant des adénopaties médiastinales dont les plus fréquents sont le cancer pulmonaire métastasé, le lymphome et la primo-infection tuberculeuse. Aucun signe ne constitue un test diagnostique de l'amylose. Seul un examen histologique d'une biopsie médiastinale, moyennant une coloration au Rouge Congo et retrouvant un aspect bi-réfringent en lumière polarisée, permet de confirmer le diagnostic d'un amyloïdome médiastinal. La biopsie des adénopathies médiastinales est le plus souvent guidée par médiastinoscopie. La biopsie ganglionnaire a été réalisée par médiastinoscopie dans notre cas ainsi que les trois cas publiés par Ridène I et al [5]. Dans certains cas, cette biopsie ne peut être réalisée que par une thoracotomie. Un cas d'amylose médiastinale a été diagnostiqué par une ponction aspiration transbronchique réalisée par l’équipe de Leiro V et al [8]. La plupart des publications rapportant un amyloïdome médiastinal isolé ont concerné une amylose AL primitive. La recherche de myélome multiple sous jacent était le plus souvent négative comme c'est le cas de notre observation [3, 4]. Une amylose AA compliquée d'un amyloïdome médiastinal isolé est exceptionnelle. Takamori S et al ont rapporté le cas d'une patiente âgée de 33 ans suivie d'une polyarthrite rhumatoïde dont l’évolution était marquée par l'installation d'une amylose médiastinale de type AA [9]. Le traitement est à la fois médical et chirurgical [7–10]. Une chimiothérapie à base de melphalan-prednisone était indiquée dans la plupart des publications en analogie avec le traitement d'une amylose systémique AL. L’évolution était marquée par une amélioration clinique et radiologique. Quant au traitement chirurgical, il est indiqué devant une masse de grande taille. Il doit être réalisé avec prudence vu la contiguïté de la masse avec des structures vasculaires rendant le risque hémorragique élevé.

Cependant, certains auteurs pensent que la résection chirurgicale est suffisante en absence d'autres localisations amyloïdes, d′autres traitements tels que le melphalan à forte dose et / ou la greffe autologue de cellules souches hématopoïétiques ne sont pas indiqués [3]. Un traitement par melphalan seul était efficace dans certains cas [7]. Ainsi, il n'existe pas de protocole thérapeutique bien codifié pour l'amylose médiastinale. Ces résultats de traitements administrés dans des cas sporadiques ne permettent pas de tirer de conclusions quant à l'efficacité de chacun d'entre eux. L’évolution était bonne dans notre cas sous traitement médical. La chirurgie était récusée vu le risque hémorragique élevé chez notre patient. Dans la publication de Ridène I et al, une atteinte ganglionnaire médiastinale pseudo-tumorale associée à une atteinte trachéo-bronchique a accéléré l'obstruction des voies aériennes [5]. Le pronostic dépend de la précocité du diagnostic positif et de la prise en charge thérapeutique.

## Conclusion

L'amylose médiastinale isolée est une maladie rare. Elle pose un problème de diagnostic positif vu la présence de plusieurs diagnostics différentiels dont l'origine néoplasique. Seuls un diagnostic précoce et un traitement adapté sont garants d'un bon pronostic.
